# Feasibility of a standardized ultrasound examination in patients with rheumatoid arthritis: a quality improvement among rheumatologists cohort

**DOI:** 10.1186/1471-2474-13-35

**Published:** 2012-03-12

**Authors:** Karen Ellegaard, Søren Torp-Pedersen, Robin Christensen, Michael Stoltenberg, Annette Hansen, Tove Lorenzen, Dorthe V Jensen, Hanne Lindegaard, Lars Juul, Henrik Røgind, Per Bülow, Stavros Chrysidis, Marcin Kowalski, Bente Danneskiold-Samsoe, Henning Bliddal

**Affiliations:** 1 The Parker Institute, Copenhagen University Hospital, Copenhagen, Denmark; 2 Sports Science and Clinical Biomechanics, University of Southern Denmark, Odense, Denmark; 3 Department of Rheumatology, Køge Hospital, Koge, Denmark; 4 Department of Internal Medicine and Rheumatology C, Gentofte University Hospital, Gentofte, Denmark; 5 Department of Rheumatology, Vejle Hospital, Vejle, Denmark; 6 Rheumatologic Clinic, Hørsholm Hospital, Hørsholm, Denmark; 7 Department of Internal Medicine C, Section of Rheumatology, Odense University Hospital, Odense, Denmark; 8 Department of Rheumatology, Copenhagen State University Hospital, Copenhagen, Denmark; 9 Department of Rheumatology H, Frederiksberg Hospital, Frederiksberg, Denmark; 10 Department of Rheumatology H, Bispebjerg Hospital, Copenhagen, Denmark; 11 Department of Rheumatology, Esbjerg Hospital, Esbjerg, Denmark; 12 Department of Rheumatology, Aalborg Hospital, Aalborg, Denmark; 13 Centre for Sensory-Motor Interaction, Aalborg University, Aalborg, Denmark; 14 Copenhagen University, Copenhagen, Denmark

**Keywords:** Ultrasonography, Rheumatoid Arthritis, Wrist

## Abstract

**Background:**

Quality improvement is important to facilitate valid patient outcomes. Standardized examination procedures may improve the validity of US.

The aim of this study was to investigate the learning progress for rheumatologists during training of US examination of the hand in patients with rheumatoid arthritis (RA).

**Methods:**

Rheumatologists with varying degrees of experience in US were instructed by skilled tutors. The program consisted of two days with hands-on training followed by personal US examinations performed in their individual clinics. Examinations were sent to the tutors for quality control. The US examinations were evaluated according to a scoring sheet containing 144 items. An acceptable examination was defined as > 80% correct scores.

**Results:**

Thirteen rheumatologists participated in the study. They included a total of 104 patients with RA. Only few of the initial examinations were scored below 80%, and as experience increased, the scores improved (*p *= 0.0004). A few participants displayed decreasing scores.

The mean time spent performing the standardized examination procedure decreased from 34 min to less than 10 minutes (*p *= 0.0001).

**Conclusion:**

With systematic hands-on training, a rheumatologist can achieve a high level of proficiency in the conduction of US examinations of the joints of the hand in patients with RA. With experience, examination time decreases, while the level of correctness is maintained. The results indicate that US may be applied as a valid measurement tool suitable for clinical practice and in both single- and multi-centre trials.

## Background

Ultrasound (US) is increasingly popular among rheumatologists for both diagnosis and monitoring of treatment of patients with rheumatoid arthritis (RA) [1-4]. US examination is assumed to be a relatively operator dependent technique. This may affect the validity of the technique [5,6] and without previous coordination of standardized examination technique, experts on performing musculoskeletal US only obtain moderate correlations of US examinations in different anatomic regions in patients with various diagnoses [6-8] and even poorer when a combination of image acquisition and interpretation is evaluated [9]. Consequently, standardized examination and interpretation procedures are required in order to improve the validity of rheumatological US examinations [10].

Educational programs in musculoskeletal US have been developed [10] and tested mostly within grey scale US [5,11-13] and focused attempts to change clinician behaviour may lead to improved outcomes [14]. Quality improvement, by educating rheumatologists to perform standardized US examination, is anticipated, while only sparse information is available as to the effect of a training program on the quality and precision of the US examination. It remains to be shown whether a learning curve might be established to describe standards for reaching and maintaining a sufficient level of US examination competence.

The ability to conduct a standardized measurement is a requirement of the OMERACT filter, which is the framework used to assess truth, discrimination, and feasibility of outcome measures in rheumatology trials and practices [15].

The one aim of the present prospective study was to evaluate the ability of a US training program to obtain standardized images of high quality in an acceptable time frame making the standardized procedure suitable for both clinical praxis and clinical trials. Thus, diagnostic utility of US and evaluation of the use of US to assess level of disease activity and monitor treatment response were not investigated in this study. A standardized examination procedure was used, which in a specialist setting has been shown to have an excellent reproducibility in patients with RA [16]. Two separate outcome measures were used as indicators of the learning process; (1) The number of examinations performed by an investigator to achieve satisfactory skills and (2) the duration of each examination.

## Methods

### Rheumatologists

In order to be enrolled in the training program it was required that the participating rheumatologist had passed two standard courses of US diagnostics arranged by the Danish Society of Diagnostic Ultrasound. All rheumatological centres in Denmark employing a rheumatologist, who has passed the required US course, were invited to participate in the project. The participating centres borrowed identical ultrasound machines with a fixed preset. Ten machines were available for the study. Ten centres agreed to participate in the study.

Table 1 shows the scanning experience and degree of supervision received by the participating rheumatologists. The scanning experience was measured in months and number of examinations and the degree of supervision was defined as 1 = none, 2 = moderate, 3 = extensive.

**Table 1:** Patient and physician characteristics at baseline

Average patients	N	Mean	SD	Minimum	Median	Maximum
Age (years)	104	54.6	14.2	20.9	56.9	82.1

DAS28, score: 0-10	100	5.1	1.2	1.5	5.1	7.4

C-Reactive Protein, mg/l	100	20	22	0	12	97

Swollen joint count, 0-28	100	8	5.25	0	6	26

Tender joint count, o-28	100	11	7.8	0	10	28

VAS global (0-100 mm)	100	58	24.1	4	60.5	100

Disease duration (years)	76	8.8	7.7	0	6	32

**Average physician**	**N**	**Mean**	**SD**	**Minimum**	**Median**	**Maximum**

US examination (minutes)	13	33.2	11.3	14	32	65

Scoring percentage	13	90	10.2	48	93	100

Scanning experience (months)	13	54	50	3	36	180

Scanning experience (number)	13	807	1475	4	250	5000

Degree of Supervision (1 to 3)*	13	1.7	0.6	1	2	3

* 1 = none, 2 = moderate, 3 = extensive

### Training program

The training program consisted of two parts. In the first part all the participating rheumatologists had an individual, two days, hands-on, training course. This part of the program took place before inclusion of patients. The rheumatologists were trained in a standardized US examination of the wrist and MCP II-V joints which has demonstrated an excellent reliability [16]. The rheumatologist performed US examinations of the wrist and MCP joints in 10 patients with RA under supervision by one of two tutors (KE and STP). The tutors had extensive parallel working experience in performing the standardized US examination (KE five years and STP more than 10 years). In the second part the tutors evaluated all the performed US examinations, which were submitted from each centre for immediate feedback by e-mail. In case of failure to reach 80% correct scores, the centre was asked to recall the patient for a repeated examination.

### RA Patients

The patients were enrolled consecutively in the rheumatological centres. Inclusion criteria were: RA diagnosed according to the American College of Rheumatology's criteria [17], Doppler activity in the wrist and/or MCP joints, ongoing therapy with methotrexate and scheduled for treatment with etanercept (only first time treatments). Exclusion criteria were treatments with other biologics within two month or lack of Doppler activity. The patients were scanned five times during the first year of etanercept therapy, or as long as they remained on this therapy. The US examinations were performed at baseline, after 2, 12, 24 and 52 weeks. The study was approved by the local ethics committee (KF 01 31 8007) and informed consent was obtained from each patient before study entry. At baseline, the patients accepted a possible delay of treatment up to one week in the case of an unacceptable baseline ultrasound examination.

### Ultrasound examination

At all centres, the US examinations were performed with a GE Logiq^® ^9 (Milwaukee, Wisconsin, USA) using a 14 MHz centre frequency linear array matrix transducer. A preset for the ultrasound examinations was installed on all machines and remained unchanged throughout the study period. The participants were allowed to make any changes to the grey scale settings in the scanning situation. The participants were not allowed to adjust the Doppler parameters except for box size and position and Doppler focus.

**Figure 1:**
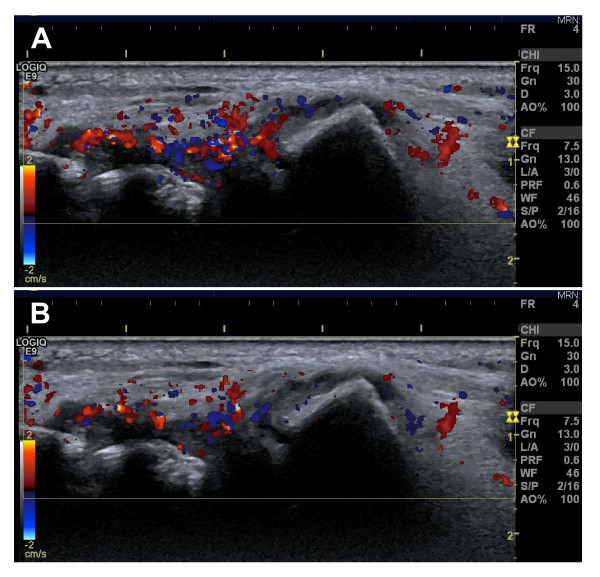
**Image of the ulnar position of the wrist in a patient with rheumatoid arthritis**. **A**: image with maximal Doppler activity. **B**: image with minimal Doppler activity. The two images are from the same cine-loop and are only a few seconds apart.

The Doppler preset was adjusted for maximum sensitivity for low flow (pulse repetition frequency of 0.4 kHz, lowest wall filter on 45 Hz, and 7.5 MHz Doppler frequency) with Doppler gain just below noise level [18]. The patient was placed sitting opposite the investigator with the hand placed on a cushion in order to keep the hand relaxed. The wrist was scanned in four positions, three dorsal and one volar position. The MCP joints were scanned in three positions, two dorsal and one volar. All scans were performed in the longitudinal plane. All participants were instructed to use generous amounts of scanning gel to avoid compression on the tissues. In all positions specific anatomic landmarks had to be present in the image to ensure the same scanning positions in all examinations (see Table 2). Once the anatomic landmarks were identified in each position, the colour Doppler was activated. While keeping the landmarks in the image, the transducer position was adjusted until the scan plane containing the most colour Doppler was found. The transducer was held in this position for a couple of heart cycles, and the image was frozen. With the cine-loop function, the frames with maximum and minimum colour Doppler activity in the synovial tissue were selected and stored (Figure 1). Subsequently, the colour Doppler was deactivated and the corresponding grey scale image containing all landmarks was obtained and stored.

**Table 2:** Landmarks at standardized ultrasound examination of wrist and MCP joints

Position	Landmarks
Wrist dorsal central	The third extensor digitorum tendon, tip of radius, carpometacarpal joint, os lunatum and os capitatum.

Wrist dorsal radial	The extensor carpi radialis longus tendon, tip of radius, carpometacarpal joint, os scaphoideum and os trapezoideum

Wrist dorsal ulnar	The middle of caput ulnae placed minimum 1 centimetre from the right side and minimum 2 from the left side of the image margins

Wrist volar central	N. medianus, tip of radius, radiocarpal and intercarpal joints

MCP* dorsal radial	Joint space, diaphysis of the metarcarpal and proximal phalanx bones. The joint space has to be placed minimum 2 centimetre from the right side and minimum 1 centimetre from the left side of the image margins

MCP* dorsal ulnar	Joint space, diaphysis of the metarcarpal and proximal phalanx bones. The joint space has to be placed minimum 2 centimetre from the right side and minimum 1 centimetre from the left side of the image margins

MCP* joint volar	Joint space and flexor tendon. The joint space has to be placed minimum 2 centimetres from the right side and minimum 1 centimetre from the left side of the image margins.

* Metacarpophalangeal joint

### Image evaluation

All US examinations were sent to the tutors for evaluation. The examinations were evaluated according to a standardized scoring system assessing the quality of all stored images. The participants received an evaluation of each US examination by e-mail. The first 50 examinations were evaluated by both tutors in order to obtain consensus. A total of 16 positions were investigated at each US examination. The total number of outcome items scored in each of the 16 positions was nine, thus a total of 144 scores was given. The scoring system was dichotomous as each outcome was assessed as accepted/not accepted. The US evaluation contained the following items: storing of all relevant images, correct annotation and image orientation (left-right), well-defined landmarks, correct focus in both grey-scale and Doppler images, presence of air or Doppler artefacts and finally correct position of the Doppler box.

In order to get the US examination approved, 80% of the items should be accepted. The cut-off point of 80% was chosen in accordance with other studies investigating learning experience in musculoskeletal US [12,13]. At each US evaluation, the duration of the examination was noted in order to evaluate the changes in the time spent.

### Statistical analyses

The data structure of the study design included clustered data (i.e, patients within clinics with repeated measures), thus the hierarchical model with continuous data was applied. Statistical analyses were based on a linear mixed model: Patients and Rheumatologists were applied as random factors when assessing associations over time. This was modelled using the Restricted Maximum Likelihood (REML) default option in SAS PROC MIXED: based on a random coefficient model in order to assess the different possible linear associations across rheumatology clinics simultaneously [19]. Random coefficient models emerge as natural mixed model extensions of simple linear regression models in a hierarchical (nested) data setup. To combine the individual study results we performed the hierarchical modelling using SAS software (version 9.2).

## Results

### Patients and rheumatologists

**Figure 2:**
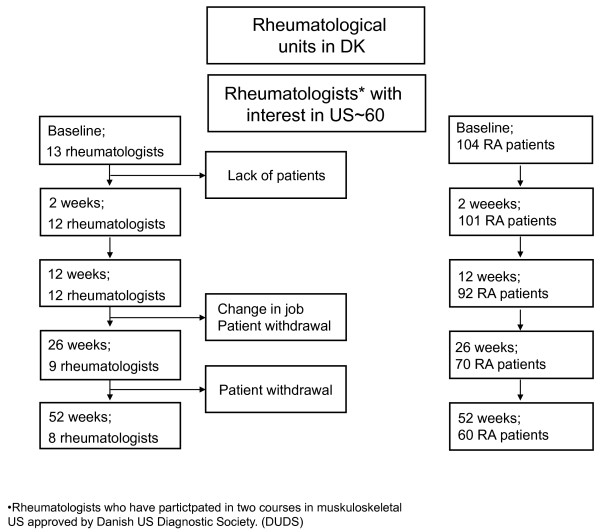
**Flow-chart of patients and participating rheumatologists**.

**Figure 3:**
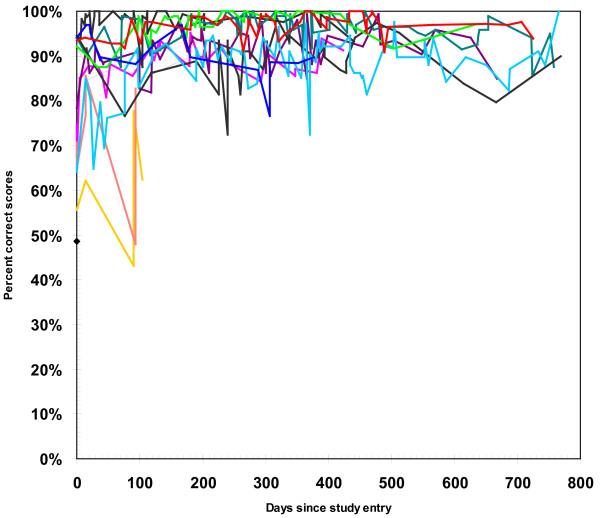
**Learning curve for the thirteen rheumatologists in the study**. Each line represents the learning progress for one rheumatologist.

There were 13 rheumatologists participating in the study. Eight of the rheumatologists (62%) succeeded in following one or more patients for one year (Figure 2). In total, 104 patients were enrolled in the study, of these 60 (58%) completed the 1 year follow-up examination (Figure 2). The mean number of patients enrolled by each rheumatologist was 8 (1-16). As presented in Table 1 the mean patient age was 54.6 (SD 14.2) years and mean disease duration was 8.8 (SD 7.7) years. Mean Disease Activity Score of 28 joints (DAS28crp) was 5.1 points (SD 1.2) with a mean swollen and tender joint count of 8 (SD 5) and 11 (SD 9), respectively and mean CRP of 20.2 mg/L (SD 21.7). As presented in Table 1 the mean scanning experience among the rheumatologists was 54 months (SD 50) and the mean number of US examinations performed before entering the study was 807 (SD 1475). Within 3 months of onset, three of the participating rheumatologists withdrew from the study; two of them due to new appointments and one because of lack of patients. The three withdrawing rheumatologists were replaced by three others, who fulfilled the inclusion criteria. Only three of the participating rheumatologists achieved a score below 80% in their initial examinations while later in the program, all scans were above the cut-off level (Figure 3).

### Learning progression

Nine of the 13 participating rheumatologists (69%) had either a consistent or slightly improved learning curve throughout the project, whereas 4 (31%) demonstrated a decreasing learning curve (Figure 3). Of these, two dropped out of the project within the first three months.

**Figure 4:**
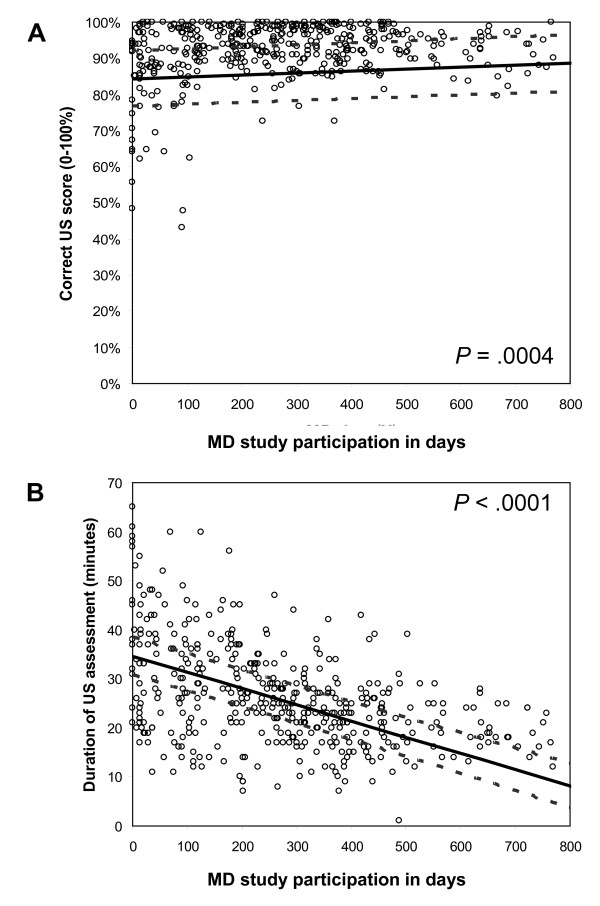
**A: Progress in the scores during the study period among the thirteen rheumatologists**. B: Time spent on one US examination during the study period for all participating rheumatologists.

As illustrated in Figure 4 most of the participating rheumatologists had above 80% correct scores from the very beginning of the study and the rheumatologists' scores increased in the study period (*p *= 0.0004; Figure 4A) corresponding to an expected improvement of 1% per hundred rheumatologist days in the study. In contrast, the time spent on an examination decreased approximately 10% per hundred rheumatologist days throughout the study period (*p *< 0.0001; Figure 4B). Relating the time spent on the US assessment with the scores achieved also showed a significant association (*p *< 0.0001). This though, was of a minor magnitude corresponding to less than 2% worsening in the US score for each of the 10 minutes saved.

## Discussion

A standardized examination procedure in patients with RA is useful for both assessment of disease activity, monitoring of treatment and in clinical trials. The results from this training program suggest that skills in standardized US examination of the hand in patients with RA can be achieved by most rheumatologists, after a short training program. Nearly, all the participating rheumatologists demonstrated scores above the preset cut-off level from the very beginning, without obvious differences due to variation in previous experience. This was also demonstrated by the lack of association between time spent on the standardized US examination and the rheumatologists' US experience. However, it must be noted that nearly all participants had a moderate to high degree of US training including several courses and two full days of hand-on experience with the present technique.

In accordance with recent results from other groups [20], this study indicates that the pitfall of US operator dependency may be avoided with a short focused training program provided that the anatomical region under investigation is sufficiently small (wrist and mcp-joints in this study). Also, bear in mind that we have only focused on ability to obtain images of a certain quality and not focused on ability to diagnose pathology.

Very low scores were only seen in a few cases and were associated with early exit from the study by some of the participants. Consequently, the study gives no answer to the obvious question whether even such rheumatologists might have achieved US examination at a higher level with further training.

The final average examination time of less than 10 minutes (Figure 4) for the standardized procedure of 16 positions, was below the examination time used in another longitudinal study in which US 17 positions were examined [21].

Despite the continuous feed-back, many participants made one persistent mistake throughout the entire study: imprecision of the standardized landmarks in the images of the MCP joints. Besides this mistake, only small flaws in the examinations were noted and with the exception of landmarks in MCP images from the analyses, the scoring level would have increased to nearly 100%. The importance of obtaining precise landmarks is recognised in US training programs [12,13] and precision of landmarks is mandatory in both longitudinal and multicentre studies to achieve comparable images.

We chose the hand as target for this training program, because the joints of the hand are frequently involved in RA [17], [22] Furthermore, it has been indicated that it is difficult to acquire satisfactory skills in US examination of the hand [5]. Thus, by choosing the hand, we avoided the bias that good learning curves were obtained by examining a simple joint.

The good results may also be attributed to our use of the same preset on all machines. This preset ensured comparable images of a relatively high quality in all patients instead of e.g. using the factory preset MSK where each exam would require some adjustments. We wished to investigate the ability to obtain reliable colour Doppler images which in our opinion demands a fixed preset. Therefore, we scored the participants' ability to correctly adjust Doppler focus and not ability to adjust Doppler gain, PRF, wall filter etc. The use of a fixed preset is a prerequisite for monitoring disease activity with Doppler and at the same time it minimises the risk of poor image quality caused by incorrect machine settings [18].

Perhaps the most important result of our study was the reduction in time spent on the examination to a feasible level for clinical practice. This result was achieved at the cost of a small reduction in score, i.e. quality, which may partly be explained by the very high scores among most of the participants from the very beginning of the study period, causing a ceiling effect, or a type of negative learning progression in a few cases. The scores did not deteriorate in a way that could indicate development of some sort of carelessness with the routine. However, the participants in trials may be more keen and accurate with supervised examinations than in the daily clinic and the results require confirmation in a clinical setting.

Standardized examination procedures improve the validity of US and make it more suitable for both clinical practice and follow-up studies. As the training program had the result that most rheumatologists achieved satisfactory skills in performing a standardized US examination it might be assumed that training will improve the quality of the US procedure and thereby the patient outcome [14]. In order to answer this question satisfactorily the effect of a standardized examination procedure on the monitoring of treatment of patients with RA must be clarified.

The present results indicate that a learning program may ensure the acquisition of standardized high quality images, which is a prerequisite for using US for making reliable diagnoses and follow-up examination e.g. according to the OMERACT filter [15]. Standardization may enable comparison of examinations performed at different institutions and make performance of multicentre trials possible.

In our study the diagnostic skills of the participating rheumatologists were not assessed and it could be assumed that skills in US scanning for a diagnostic purpose will demand more training to achieve a satisfactory level.

## Conclusion

In conclusion, this study shows that skills in standardized US examinations of the hand are relatively easy to obtain for most rheumatologists even with limited US experience.

## Authors' contributions

KE: supervised in study design, collected data, made the manuscript; STP: supervised in study design and critical review of the manuscript; RC: statistical analyses; MS: collected data, critical review of the manuscript; AH: collected data, critical review of the manuscript; TL: collected data, critical review of the manuscript; DVJ: collected data, critical review of the manuscript; HL: collected data, critical review of the manuscript; LJ: collected data, critical review of the manuscript, HR: collected data, critical review of the manuscript; PB: collected data, critical review of the manuscript; SC: collected data, critical review of the manuscript; MC: collected data, critical review of the manuscript; BDS: critical review of the manuscript; HB:conceived the study and critical review of the manuscript. All authors read and approved the final manuscript.

## Disclosure

The EURA study was sponsored by the Oak Foundation and a restricted grant from Wyeth A/S Denmark. KE and STP have received fees for educational activities sponsored by Wyeth. None of the authors are employed by Wyeth.

## Pre-publication history

The pre-publication history for this paper can be accessed here:

http://www.biomedcentral.com/1471-2474/13/35/prepub
